# Supportive Care: The “Keystone” of Modern Oncology Practice

**DOI:** 10.3390/cancers15153860

**Published:** 2023-07-29

**Authors:** Florian Scotté, Amy Taylor, Andrew Davies

**Affiliations:** 1 Gustave Roussy, 94805 Villejuif, France; 2 Our Lady’s Hospice & Care Services, D6W RY72 Dublin, Ireland; 3 School of Medicine, Trinity College Dublin, D02 PN40 Dublin, Ireland; 4 School of Medicine, University College Dublin, D04 V1W8 Dublin, Ireland

**Keywords:** supportive care, cancer, multidisciplinary

## Abstract

**Simple Summary:**

This article provides an overview of supportive care in cancer, a service that supports people living with and beyond cancer through the prevention and management of the side effects of their disease and its treatment. Supportive care is relevant throughout the entire cancer journey from diagnosis, through treatment, to care after treatment. This article discusses the definition of supportive care and explores relevant concepts, including its relationship with palliative care. It also discusses models of care, “core” service elements, evidence of the benefits of supportive care (including economic benefits), and future directions and challenges in providing supportive care for all patients diagnosed with cancer.

**Abstract:**

The Multinational Association of Supportive Care in Cancer (MASCC) defines supportive care as “the prevention and management of the adverse effects of cancer and its treatment. This includes management of physical and psychological symptoms and side effects across the continuum of the cancer journey from diagnosis through treatment to post-treatment care. Supportive care aims to improve the quality of rehabilitation, secondary cancer prevention, survivorship, and end-of-life care”. This article will provide an overview of modern supportive care in cancer, discussing its definition, its relationship with palliative care, models of care, “core” service elements (multi-professional/multidisciplinary involvement), the evidence that supportive care improves morbidity, quality of life, and mortality in various groups of patients with cancer, and the health economic benefits of supportive care. The article will also discuss the current and future challenges to providing optimal supportive care to all oncology patients.

## 1. Introduction

The term “supportive care” has been used to describe various types of intervention within medicine, especially oncology [1]. For example, it has been used to describe intensive medical treatment (e.g., major organ support), “palliative” anticancer treatment, pain and symptom control (i.e., cancer-related, cancer treatment-related), psychosocial support, and even a lack of specific treatment [2]. It has also been used as a euphemism for palliative care, which is discussed in detail below [3]. However, supportive care (in cancer) is none of these things, although pain and symptom control, psychosocial support, and palliative care are important components of “true” supportive care.

The aim of this article is to provide an overview of supportive care, which has come to be accepted as an “indispensable component” of modern oncology [4,5]. The objectives are to define supportive care, discuss models of supportive care with their rationale and evidence base, and explore differences and complementarities between supportive care and palliative care. The manuscript highlights the activities of the Multinational Association of Supportive Care in Cancer (MASCC), which is the leading international organisation for professionals and other people working within supportive care in oncology (e.g., patient advocates and industry partners) [6]. 

## 2. Definition of Supportive Care

The medical literature contains a number of different definitions for supportive care, which reflect the different types of “supportive care” interventions (see above) [1]. MASCC defines supportive care as “the prevention and management of the adverse effects of cancer and its treatment. This includes management of physical and psychological symptoms and side effects across the continuum of the cancer journey from diagnosis through treatment to post-treatment care. Supportive care aims to improve the quality of rehabilitation, secondary cancer prevention, survivorship, and end-of-life care” [6]. Moreover, MASCC states that “supportive care makes excellent cancer care possible” (Box 1) [6].

Box 1 **Principles of supportive care (Multinational Association of Supportive Care in Cancer) [6].**

❖ Supportive care aims to maintain (or improve) quality of life and to ensure that people with cancer can achieve the maximum benefit from their anticancer treatment;
❖ Supportive care is relevant throughout the continuum of the cancer experience, from diagnosis through treatment to post-treatment care (and encompasses cancer survivorship and palliative and end-of-life care);
❖ Supportive care involves a coordinated, person-centric, and holistic (whole-person) approach, which should be guided by the individual’s preferences and should include the appropriate support of their family and friends;
❖ Supportive care (as outlined) is a basic right for all people with cancer, irrespective of their personal circumstances, their type of cancer, their stage of cancer, or their anticancer treatment. It should be available in all cancer centres, and other medical facilities that routinely manage people with cancer.

In comparison, the National Cancer Institute (NCI) (USA) defines supportive care as “care given to improve the quality of life of people who have an illness or disease by preventing or treating, as early as possible, the symptoms of the disease and the side effects caused by treatment of the disease. Supportive care includes physical, psychological, social, and spiritual support for patients and their families. There are many types of supportive care. Examples include pain management, nutritional support, counselling, exercise, music therapy, meditation, and palliative care. Supportive care may be given with other treatments from the time of diagnosis until the end of life” [7].

“Best supportive care” is sometimes used interchangeably with supportive care [2,8], although this is somewhat of a misnomer, since the underlying principles of supportive care involve providing optimal (“best”) care to patients and their families [6]. Of note, “best supportive care” was the control arm in many historical oncology treatment research studies, although this often involved no specific care rather than predefined supportive care [2]. In response to this lack of care, an expert group developed consensus guidelines for standards for best supportive care in clinical trials (in patients with advanced cancer) [9]:

- Multidisciplinary care:
  - Patients should have access to palliative care specialists while receiving anticancer therapy;
  - Patients should have access to high-quality nursing, social work support, financial counselling, and spiritual counselling;
  - Cooperative groups and institutional review boards should encourage standard processes to educate patients so that they understand the goals of anticancer therapy, the importance of symptom assessment, and the role of symptom management within the clinical trial.
- Documentation:
  - For trials with a significant best supportive care component, institutional review boards should review trial protocols for documentation of supportive care interventions;
  - For trials with a significant best supportive care component, the delivery of supportive care within a clinical trial should be documented in a standard way for all patients on that trial;
  - Journal editors should ask for a clear description of what best supportive care entailed in reports of trials with a significant best supportive care component.
- Symptom assessment:
  - Symptoms should be assessed at baseline and regularly throughout the trial;
  - Symptoms should be assessed using concise, globally accessible, and validated tools.
- In trials in which patients are enrolled in a best supportive care group vs. an intervention group, the intervals between symptom assessments should be identical in both groups;
- Symptom management:
  - Symptom control should be conducted in line with evidence-based guidelines;
  - Clinical trial protocols should encourage guideline-based symptom control.

“Enhanced supportive care” refers to an initiative by NHS England, which was “developed through recognition of what specialist palliative care can offer but also from recognition of the barriers to achieving earlier involvement of palliative care expertise within the cancer treatment continuum” [10] (p. 4). This initiative was focused on so-called “early” palliative care rather than predefined supportive care [11], although some services did adopt a broader approach. Indeed, many of the patients treated had advanced cancer, and there was a greater focus on the management of cancer-related problems than of cancer treatment-related toxicities. The relationship between palliative care, “early” palliative care, and supportive care is discussed in more detail beneath. 

Recently, it has been suggested that the term supportive care be replaced by the term “supportive oncology” because of the latter’s misappropriation to describe specialist palliative care services [12]. Table 1 provides a summary of such terminologies relevant to “supportive care”.

## 3. Models of Supportive Care

**Figure 1:**
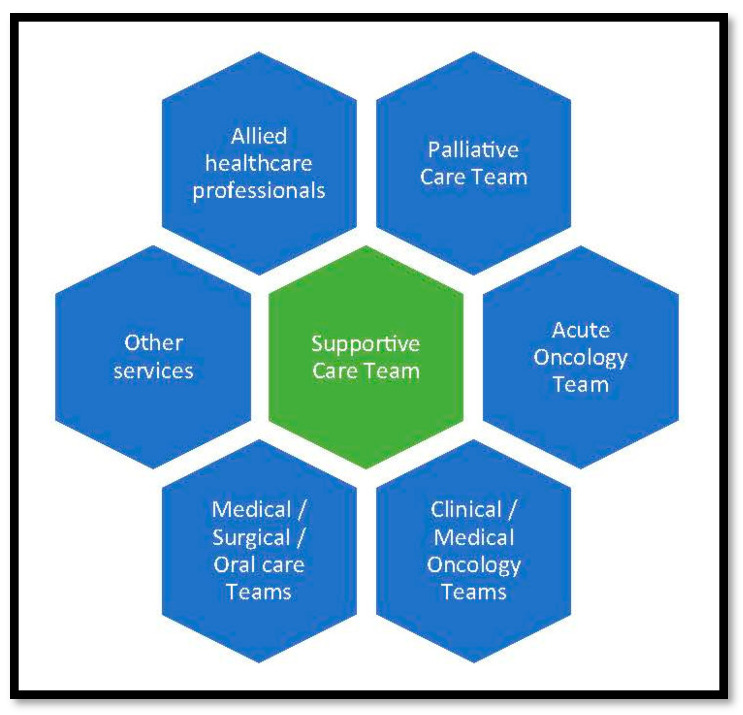
The “extended” supportive care team [5]. “Other services” include (but are not confined to) psychology, social work, and pastoral care services. “Allied healthcare professionals” include (but are not confined to) physiotherapists, occupational therapists, dieticians, and radiographers.

Supportive care encompasses the whole cancer experience and so necessitates the involvement of most clinical specialties and of many nonclinical services (Figure 1) [5,16]. Indeed, modern supportive care cannot be provided by a single clinical specialty (or single professional group). However, as with other cancer multidisciplinary teams, there needs to be a dedicated “core team” to manage everyday problems (e.g., oncology specialists and palliative care specialists), with timely input from the “extended team” as and when the need arises (e.g., organ-specific medical specialists, pain specialists, psychologists, and allied healthcare professionals). Allied healthcare professionals refer to a wide range of non-nursing/nonmedical specialists, including physiotherapists, occupational therapists, dietitians, and radiographers. Importantly, the core team need specific knowledge and training in the major supportive care interventions.

Supportive care services provide a wide range of interventions including (but not limited to):

- Management of cancer-related symptoms/problems:
  - Pain;
  - Other symptoms.
- Management of cancer treatment-related symptoms/problems:
  - Prophylaxis, e.g., antiemetics to prevent chemotherapy-induced nausea and vomiting;
  - Treatment.
- Coordinating management of co-morbidities with other specialties;
- Psychological support:
  - Patient;
  - Carers (children).
- Nutritional support;
- Prehabilitation;
- Rehabilitation;
- Social care:
  - Advocacy;
  - “Financial toxicity”.
- Palliative care:
  - “Early palliative care”;
  - End-of-life care/bereavement care.
- Survivorship care;
- Integrative therapies.

Supportive care teams should be available in inpatient and outpatient settings, with close cooperation with the patient’s primary care team and specialist input from other relevant health and social care services as required. Historically, there have been problems related to a lack of coordination, poor communication (between professionals and patients and among different groups of professionals), poor continuity of care, and non-standardised approaches to care. In response, “new” professional roles have been developed such as patient “navigators” (i.e., healthcare professionals or volunteers who assist patients and their families in decision making, accessing services, and overcoming health- and social care barriers) [17], and “advanced” practitioners in supportive care (e.g., nurse practitioners and physician assistants) [18,19]. Furthermore, digital health interventions are increasingly being utilised to support both professionals and patients and their families (see below) [20].

**Figure 2:**
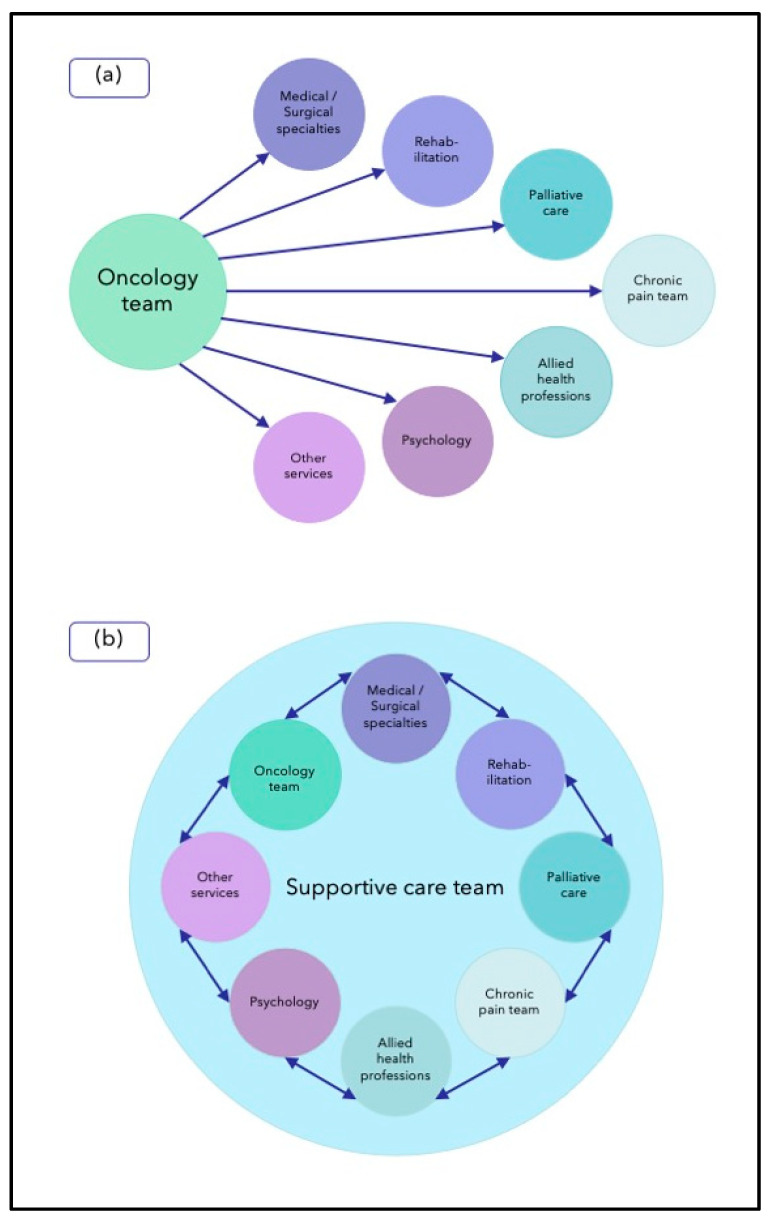
Comparison of service models: (**a**) services provide supportive care in isolation; (**b**) supportive care team provides services within an integrated care model.

Currently, supportive care services vary widely in terms of their personnel and routine functions [21]. Some services are somewhat exclusive, with the core team only able to undertake certain functions and needing to refer to other (independent) teams/services for specific interventions. Other services are much more inclusive, adopting the so-called “integrated supportive care model” [21]; this approach involves historically independent teams/services coming together as a unified supportive care team/service (Box 2; Figure 2).

Box 2 Unified supportive care model (adapted from Hui et al. [21]). * Dependent on available resources.

❖ Integrated supportive care team/service (see Figure 1);
❖ Consolidated leadership;
❖ Collaborative teamwork;
❖ Streamlined care (as a result of collaborative teamwork);
❖ Universal referral of cancer patients; *
❖ Systematic screening—to assess unmet supportive care needs, guide supportive care interventions, and facilitate timely supportive care interventions;
❖ Tailored specialist involvement (as a result of systematic screening);
❖ Consistent messaging (as a result of collaborative teamwork)

Supportive care service models must be flexible to be applicable to all people living with and beyond cancer. Thus, the ageing population (with frequent comorbidities), the increasing incidence of cancer, the increasing number of long-term “cancer survivors” (with chronic problems), and the emergence of new interventions (with unique toxicities) will all increase the pressure on existing supportive care services in the coming years. Moreover, it is likely that models and services will need to evolve with greater input from, for example, organ-specific specialists and gerontologists.

As discussed, digital health interventions have been identified as a means of improving service efficiency and patient outcomes [6]. Digital health, telehealth, and eHealth are interchangeable terms, defined as “the provision of healthcare services supported by telecommunications or digital technology to improve or support healthcare services” [22] (p. 4590). Digital health interventions encompass patient monitoring, symptom management, and self-management, which are all relevant to supportive care and have the potential to improve service efficiency [22]. There is evidence that collecting electronic patient-reported outcomes and digital supportive care interventions (such as psychotherapeutic, mindfulness, exercise, and rehabilitation programmes) improve distress, symptoms, and quality of life in people living with cancer [22,23]. In addition, remote monitoring can increase treatment adherence (and efficacy) and allow for the early detection of problems or relapse [22] in an acceptable way for patients and families [24]. There has also been interest in utilising machine learning to predict future outcomes [25], which could help better target health- and social care resources. However, digital services must complement and not replace direct in-person care. Future research is required to determine appropriate strategies to respond to outputs from digital interventions to achieve stakeholder engagement and to support implementation into routine practice.

Information sharing has been identified as a particular supportive care need, and when lacking, this is a cause of considerable distress for patients and their carers [26]. Supportive care models should incorporate the provision of easily accessible, understandable (simple language), quality information tailored to the different stages of the disease trajectory. Additionally, services must recognise relevant ethnic, cultural, and economic sensitivities. A way to effectively meet these needs is by involving patients and carers in service development.

## 4. Rationale and Evidence for Supportive Care

The primary reason for providing supportive care involves preventing and managing the morbidity relating to the cancer and/or the cancer treatment. In other words, it is about improving the patient’s quality of life (and that of their carers). Additionally, there are potential survival benefits as adequate prevention and management of these problems may prevent premature mortality (e.g., sepsis related to neutropenia). An equally important reason for providing supportive care involves facilitating anticancer treatment regimens. Minimising the adverse effects from anticancer treatments will lead to better adherence and improve the relative dose intensity for chemotherapy (i.e., the ratio of the dose intensity delivered relative to the standard dose intensity) and equivalent parameters for other anticancer treatments [27], resulting in better outcomes (i.e., prolonged survival, permanent remission). Supportive care in cancer can, therefore, directly improve quality of life and indirectly improve quantity of life.

Numerous studies have demonstrated the existence of a range of unmet needs throughout the entire cancer journey and the negative impact such unmet issues have on the lives of patients and their families [26,28,29]. Although specific unmet needs can differ among cancer types [28], the breadth of issues across all domains (i.e., physical, psychological, social, spiritual, informational, and practical) is common to all cancers [26,28,29]. The principles of supportive care involve addressing all such unmet needs, through ongoing holistic assessment and appropriate interventions from relevant members of the extended supportive care team. Recently, there has been greater recognition of the issue of financial toxicity for patients and their families as a result of the direct costs of anticancer treatment and, equally, the indirect costs associated with a cancer diagnosis and cancer treatment (e.g., loss of income and travel costs) [26,29].

Supportive care services are increasingly involved in the management of long-term cancer survivors. The NCI defines cancer survivors as individuals “from the time of diagnosis through the balance of life”, which includes “those living with cancer and those free of cancer” [30]. Traditionally, supportive care services have focused on patients living with (active) cancer rather than patients free of cancer (and indeed patients on long-term maintenance anticancer treatments). However, the recognition that the latter groups of cancer patients have high unmet needs (in all domains) and often somewhat unique problems (e.g., chronic problems relating to previous treatment) has led to the development of tailored supportive care services. The need for such services is only likely to increase because of the expected increase in the numbers of long-term cancer survivors.

Various models of supportive care have been formally assessed, such as multidisciplinary services aligned to oncology [31,32] and also nurse-led services [33]. The evidence suggests that supportive care services can improve symptom control [31], improve quality of life [31], reduce emergency room attendances [33,34], reduce hospital admissions [32,34], shorten hospital admissions [31,32], reduce chemotherapy deferrals [31], reduce 30-day mortality after chemotherapy [31], improve overall survival [31], and reduce healthcare-related costs [32,33,34]. Nevertheless, further research is required to confirm these findings and to identify the optimal models for supportive care throughout the cancer continuum.

It should be noted that while there is limited evidence to support models of supportive care, there is a wealth of evidence to support the use of interventions to manage the specific problems encountered within supportive care [35]. For example, chemotherapy-induced nausea and vomiting was previously a major issue, resulting in significant morbidity and interruption or discontinuation of chemotherapy (with a negative impact on tumour response and overall survival). However, the development of new classes of drugs (e.g., 5HT3 antagonists and NK1 antagonists), and especially the development of evidence-based prescribing guidelines, has led to a dramatic reduction in troublesome nausea and vomiting. Indeed, in 2014, the American Society for Clinical Oncology (ASCO) identified progress in antiemetics as one of the five best advances in modern oncology over the previous 50 years, since it hugely improved the experiences of patients undergoing anticancer treatment, reducing hospital admissions, increasing treatment completion rates, and improving treatment outcomes [36].

Increasing pressure on the limited healthcare resources has necessitated formally assessing the health economic benefits of supportive care services. As discussed above, such services have been shown to produce a number of positive clinical benefits (e.g., reduced emergency room attendances and reduced hospital admissions) which, in turn, have resulted in cost savings within secondary and tertiary care settings. Indeed, these health economic assessments further support the argument for allocating or reallocating resources towards supportive care service development [32,33]. Additionally, social return on investment (a cost–benefit analysis that evaluates the social, economic, and environmental impacts of interventions [37]) has shown that an optimally coordinated supportive care service leads to wider financial benefit that increases cumulatively with time from cancer diagnosis [38].

## 5. Supportive Care Versus Palliative Care

The International Association for Hospice and Palliative Care (IAHPC) define palliative care as “the active holistic care of individuals across all ages with serious health-related suffering due to severe illness and especially of those near the end of life. It aims to improve the quality of life of patients, their families and their caregivers” [13] (p. 761). The definition is supported by a series of additional characteristics:

- Includes prevention, early identification, comprehensive assessment, and management of physical issues, such as pain and other distressing symptoms, psychological distress, spiritual distress, and social needs. Whenever possible, these interventions must be evidence based;
- Provides support to help patients live as fully as possible until death by facilitating effective communication, helping them and their families determine goals of care;
- Is applicable throughout the course of an illness, according to the patient’s needs;
- Is provided in conjunction with disease-modifying therapies whenever needed;
- May positively influence the course of illness;
- Intends neither to hasten nor to postpone death, affirms life, and recognises dying as a natural process;
- Provides support to the family and caregivers during the patient’s illness, and in their own bereavement;
- Is delivered recognising and respecting the cultural values and beliefs of the patient and family;
- Is applicable throughout all healthcare settings (place of residence and institutions) and in all levels (primary to tertiary);
- Can be provided by professionals with basic palliative care training;
- Requires specialist palliative care with a multiprofessional team for referral of complex cases.

As discussed, supportive care has been used as a synonym for palliative care (especially “early palliative care”). However, although palliative care is an integral component of supportive care, supportive care is much more than palliative care and involves input from a range of specialist teams and services (Figure 1) [5]. Importantly, many specialist palliative care teams have limited knowledge and experience in managing the adverse effects of anticancer treatment, and especially the newer immunotherapies or targeted therapies [39]. Moreover, the interventions they use to manage the complications of advanced disease are often completely different to the interventions used to manage the complications of anticancer treatment (e.g., antiemetic drugs for nausea and vomiting) [40,41].

Nevertheless, a number of specialist palliative care services have rebranded themselves as supportive care services (or supportive and palliative care services) because of the negative public and professional perceptions concerning the term palliative care, i.e., that palliative care is synonymous with end-of-life care [42]. Indeed, research suggests that oncology healthcare professionals are reluctant to refer to palliative care services because of concerns about causing patients distress and/or reducing patients’ hope (and that of their families) [42]. Importantly, other research suggests that such a name change can lead to an increase in referrals, particularly referral of patients on active treatment and at an earlier stage in their cancer journey than beforehand [43]. It should be noted that some supportive and palliative care teams do provide both palliative care interventions and predefined supportive care interventions under a single service.

Of note, the European Society for Medical Oncology (ESMO) Supportive and Palliative Care Faculty have proposed the term “patient-centred care” to include both supportive care, as defined by MASCC, and palliative care, as defined by the World Health Organisation [4], i.e., “palliative care is an approach that improves the quality of life of patients (adults and children) and their families who are facing problems associated with life-threatening illness. It prevents and relieves suffering through the early identification, correct assessment and treatment of pain and other problems, whether physical, psychosocial or spiritual” [44]. ESMO adopted the term in response to the recognition of the increasing gap between cancer patients’ needs and their care, advocating that organisations should deliver related services collaboratively by involving all appropriate professionals and interventions [4].

## 6. Multinational Association for Supportive Care in Cancer (MASCC)

MASCC has been the pre-eminent international organisation for professionals involved in supportive care in cancer for the last 30 years [6,16]. It has members in more than 70 countries (Africa, Asia, Australia/Oceania, Europe, North America, and South America) and from almost all oncology-related disciplines (clinical, nonclinical, and basic sciences). It is affiliated with several national supportive care organisations (India, Italy, Japan, Korea, Portugal, Russia, Serbia, and France/French-speaking countries), and has partnerships with the International Society of Oral Oncology and a number of major international oncology organisations (e.g., Oncology Nursing Society, ESMO).

MASCC’s mission “is to continually improve the supportive care of people with cancer”, and it does this through its study groups and a variety of educational and research initiatives [6]. Currently, there are 16 study groups (and four subgroups), which range in remit from specific groups of patients (e.g., geriatric study group and paediatric study group), broad topic areas/issues (e.g., oral care study group and immune-oncology subgroup), specific symptoms/issues (e.g., antiemetic study group and mucositis study group), and generic topics (e.g., education study group and digital health subgroup) [6]. One of the major outputs of the study groups are the evidence-based guidelines, which are often undertaken with major international oncology organisations (and which are continually updated) [45,46]. MASCC organises an annual research meeting, as well as regular webinars on current issues within supportive care. The MASCC journal is *Supportive Care in Cancer*, and there is a MASCC Textbook of Cancer Care and Survivorship [35].

Recently, MASCC introduced an accreditation scheme for cancer centres (“MASCC-Designated Centres of Excellence in Supportive Care in Cancer”); accreditation requires a comprehensive supportive care team/service, integration of the team/service within the oncology centre, use of evidence-based supportive care guidelines and interventions, and a commitment to supportive care education and research [6]. The ultimate objectives of the scheme are to support the development of new supportive care services, support the evolution of existing supportive care services, and improve the accessibility, availability, and quality of supportive care services for all patients with cancer and their caregivers worldwide.

## 7. Future Directions

The practice of oncology is constantly evolving, and supportive care services will need to adapt and evolve to maintain their relevance. Changes in population demographics (i.e., an “ageing” population) will mean more cases of cancer, and more patients with significant co-morbidities. Thus, there will need to be investment in supportive care services to meet this increased demand, and even greater co-operation with gerontology and other medical specialties (to optimise management of the co-morbidities in order to maximise the anticancer treatment opportunities).

Current anticancer treatments are leading to more patients “living with cancer” and more patients “living beyond cancer”; many of these patients will have ongoing problems that require specialist treatment. “Conventional” treatments may be relatively unsuitable for some of these cohorts of patients. For example, opioids are important analgesics for patients with acute pain, especially patients with advanced cancer. However, the role of opioids in chronic pain, which is a common problem in patients following anticancer treatment, is unclear [47]. Furthermore, there are concerns regarding the long-term effects of opioids on the endocrine system (hypogonadism), immune system, respiratory system, and central nervous system, as well as the perennial risk of drug misuse and abuse [48]. As a result of issues such as these, there will be an increase in demand for supportive care services from current patients, as well as from future patients. Importantly, additional research is required to determine the optimal management of these ongoing problems. It is also likely that novel anticancer treatments will generate unique toxicities, which will require novel supportive care interventions (necessitating additional research, education, and training). These novel anticancer treatments should result in even longer survival, which would again increase the number of cancer patients with chronic problems (as well as the number of cancer patients requiring supportive care).

Importantly, the extension of specialist supportive care services must also be supported by the education and training of the wider oncology workforce in the principles of supportive care. Specialist services will not be able to manage all of the supportive care needs of individuals living with and beyond cancer. Primary care providers will remain the constant factor in most patient journeys, and they should receive training and updates on the principles of supportive care. Furthermore, supportive care should be a core component of all oncology-related undergraduate and postgraduate curricula. Indeed, it is included in the ESMO/ASCO Recommendations for a Global Curriculum in Medical Oncology, which specify that trainees should develop knowledge and understanding of the symptoms of cancer, side effects of treatments, and relevant supportive care guidelines, including prevention and management strategies [49].

## 8. Conclusions

Supportive care is an “indispensable component” of modern oncology and is associated with improved quality of life, improved tolerability of anticancer treatments, improved survival (as a result of improved adherence with anticancer treatments), and systemic health economic benefits. The service model should be coordinated and integrated and involve a range of relevant healthcare professions and disciplines. All cancer centres should have properly resourced supportive care teams and services, and all cancer patients should have access to these services irrespective of their stage or phase of disease.

## Figures and Tables

**Table 1:** Summary of terminologies relevant to “supportive care”.

Terminology	Definition
Supportive care	“The prevention and management of the adverse effects of cancer and its treatment. This includes management of physical and psychological symptoms and side effects across the continuum of the cancer journey from diagnosis through treatment to post-treatment care. Supportive care aims to improve the quality of rehabilitation, secondary cancer prevention, survivorship, and end-of-life care” [6].
Palliative care	“The active holistic care of individuals across all ages with serious health-related suffering due to severe illness and especially of those near the end of life. It aims to improve the quality of life of patients, their families and their caregivers” [13] (p. 761).
Early palliative care	“Palliative care treatments applied early in the course of a life-threatening disease…In cases of advanced cancer, early palliative care is provided alongside active disease treatment such as chemotherapy or radiotherapy” [11] (p. 7).
Timely palliative care	“Early palliative care personalised around patients’ needs and delivered at the optimal time and setting” [14] (p. 3).
Best supportive care	No agreed definition, although consensus guidelines are available for best supportive care in clinical trials in advanced cancer [9].
Enhanced supportive care	An initiative by NHS England, which was “developed through recognition of what specialist palliative care can offer, but also from recognition of the barriers to achieving earlier involvement of palliative care expertise within the cancer treatment continuum” [10] (p. 4). (Enhanced supportive care is synonymous with early palliative care).
Supportive oncology	“Those aspects of medical care concerned with the physical, psychosocial, and spiritual issues faced by persons with cancer, their families, their communities, and their health-care providers. In this context, supportive oncology describes both those interventions used to support patients who experience adverse effects caused by antineoplastic therapies and those interventions now considered under the broad rubric of palliative care” [15] (p. xii). (Supportive oncology is synonymous with supportive care).

## Data Availability

The data can be shared up on request.
